# Enhancing Gypsum Plaster with Encapsulated Fischer–Tropsch Paraffin Wax as a Phase-Change Additive for Broad-Range Thermal Energy Storage

**DOI:** 10.3390/polym18091111

**Published:** 2026-04-30

**Authors:** Denis Voronin, Ekaterina Smirnova, Nataliya Demikhova, Adeliya Sayfutdinova, Dmitry Kopitsyn, Rawil Fakhrullin, Vladimir Vinokurov, Anna Stavitskaya

**Affiliations:** 1 Department of Physical and Colloid Chemistry, National University of Oil and Gas “Gubkin University”, 119991 Moscow, Russiastavitsko@mail.ru (A.S.); 2 Institute of Fundamental Medicine and Biology, Kazan Federal University, Kreml Uramı 18, 420008 Kazan, Republic of Tatarstan, Russia; 3 Biological Institute, Tomsk State University, 36 Lenin Ave., 634050 Tomsk, Russia

**Keywords:** Fischer–Tropsch wax, latent heat storage, phase-change materials, paraffin wax, energy efficiency, thermal regulation

## Abstract

Paraffins are attractive as phase-change materials (PCMs) due to their high latent heat capacity and adjustable phase transition temperatures. However, the individual high-purity paraffins, especially the long-chain ones, are labor-intensive and costly to produce and capable of storing and releasing latent heat only within a limited temperature range. Herein, we demonstrate the feasibility of a high-purity paraffin wax fraction (C13–C49) obtained via the Fischer–Tropsch (FT) process as a versatile latent heat storage additive within a wide range of phase transition temperatures (8.1–98.2 °C). To avoid the leakage, the FT wax was encapsulated via nanoemulsion interfacial polymerization of melamine formaldehyde (MF) shells with various core-to-monomer and melamine/formaldehyde ratios. Differential scanning calorimetry revealed that the latent heat storage capacity of the FT/MF capsules was 104.5–163.4 J/g depending on the FT loading efficiency, with the heat storage and release range of −0.7–100.2 °C and −9.8–85.8 °C, respectively. The capsules were tested as a thermoregulating additive to commercially available gypsum plaster. Unlike employment of the additives based on individual paraffins, the addition of FT/MF capsules led to a smooth reduction in heating/cooling rates of plaster layers in an extended temperature range. This makes FT/MF capsules a promising and versatile additive for a diversity of thermal energy storage applications.

## 1. Introduction

Improving the energy efficiency of buildings is a key challenge in the context of sustainable development and reducing anthropogenic environmental impact. In this regard, phase-change materials (PCMs), capable of accumulating, storing, and releasing significant amounts of thermal energy during reversible melting–crystallization processes, are of significant interest. The integration of microencapsulated PCMs into construction materials allows for the passive stabilization of indoor temperatures by smoothing daily fluctuations, which directly leads to reduced energy consumption for heating and cooling [1,2].

Considering organic PCMs, paraffins are among the most studied due to their high specific latent heat of phase transition, adjustable melting point, lack of sub-cooling, and chemical stability [3,4]. However, the use of individual, highly purified n-alkanes to achieve a specific phase-change temperature is associated with significant expenses. A more economical alternative is technical paraffin fractions [5]. In this context, paraffin fractions obtained via the Fischer–Tropsch process (FT) are of particular interest. This process involves the catalytic conversion of synthesis gas into a mixture of linear hydrocarbons. Subsequent refining of this synthetic crude oil, specifically the hydrocracking of heavy waxes followed by vacuum distillation, allows for the isolation of narrow paraffin fractions with a specified carbon number range. Key advantages of such paraffins are their high purity with a predominance of linear n-alkanes; a broad and tunable phase-change temperature range, which is an advantage for thermal regulation under varying temperature conditions; and competitive cost, as production is independent of traditional oil refining [6,7].

The main technological barrier for using paraffins as latent heat storage additives is the leakage in the liquid state. Encapsulation into micro- and nanoscale capsules through the formation of a durable polymer shell around a paraffin core appears to be an efficient approach to deal with the leakage issue, prevent paraffin interaction with a surrounding medium, and enhance a surface area to improve the heat transfer [3,8]. The encapsulation includes various approaches, which can be roughly grouped into physical, chemical, and hybrid methods. Regarding wide paraffin fractions containing high molecular weight components with considerable melting points, in situ polymerization of amino-formaldehyde resin appears a versatile approach, as it utilizes a condensation of water-soluble prepolymer at the interface of premixed paraffin emulsion or suspension to form a robust polymer shell under mild conditions [9,10,11,12,13]. Unlike the interfacial polymerization of polyurethane/polyurea or polymethyl methacrylate shells involving the introduction of hydrophobic monomers to the oil phase, in situ polymerization allows for avoiding side reactions, which can be initiated by the extensive system heating required to keep the paraffin permanently melted during emulsification. In particular, melamine (1, 3, 5-triamino-2, 4, 6-triazine) formaldehyde (MF) resins possess good mechanical strength, high thermal stability, and excellent chemical and fire resistance due to their highly cross-linked structure [14]. Furthermore, the properties of the MF resins can be adjusted through the variation in the melamine/formaldehyde molar ratio [15,16].

To date, the encapsulation of PCMs via in situ polymerization of MF capsule shells has been widely studied, and the mechanisms and parameters of MF shell condensation are well-established [17,18]. Nevertheless, the encapsulation of FT wax within the MF shell was not reported previously. Mochane and Luyt reported the microencapsulation of FT wax via suspension polymerization of polystyrene shell [19]. However, the loading efficiency was only 20–30 wt.%, which corresponded to the latent heat capacity of 40 J/g. Taking into account that the microcapsules tend to count for about 10–20 wt.% as a thermoregulating additive, this is relatively low. In earlier studies, FT waxes were employed as PCMs for the preparation of latent heat storage composites by embedding them into polymer matrices, like various styrene derivatives, polyethylene, and polypropylene [20,21,22]. On the one hand, the blending of FT wax with polymers effectively suppressed its leakage. On the other hand, this approach is limited to polymer matrices immiscible with paraffins, which cannot directly replace conventional building materials.

Thus, the aim of this study was to develop an efficient method for FT wax encapsulation and evaluate the feasibility of encapsulated FT wax as a thermoregulating additive to commercially available gypsum plaster. To do this, nanosuspension of FT wax was prepared and encapsulated within an MF shell via in situ polymerization with various core-to-monomer ratios. The thermoregulating properties and stability of the capsules were evaluated depending on the FT/MF capsule composition. The capsules with an optimal combination of structural properties, latent heat capacity, and stability were added to the gypsum plaster. The effect of FT/MF capsules on the heating and cooling rate of the plaster layer was evaluated depending on the layer thickness and heating rate.

## 2. Materials and Methods

### 2.1. Materials

The paraffin wax obtained via the Fischer–Tropsch process (FT wax) was provided by Platov South-Russian State Polytechnic University (NPI) (Novocherkassk, Russia). The FT wax met the following manufacturer specifications: mass fraction of mechanical impurities ≤ 0.05%, sulfur content ≤ 1 ppm, and absence of detectable water or water-soluble acids and bases.

Sodium dodecyl sulfate (SDS, ≥99.0%), Triton X-100 (laboratory grade), melamine (99%), formaldehyde (37% aqueous solution), and triethanolamine (TEA, 97%) were purchased from Sigma-Aldrich (Darmstadt, Germany). Polyvinyl alcohol (PVA, *n* = 1700) was purchased from TCI (Tokyo, Japan).

Deionized water with the specific resistivity no lower than 18.2 MΩ∙cm was used as an aqueous medium in all synthesis and rinsing procedures.

### 2.2. Methods

#### 2.2.1. Preparation of FT Wax Emulsion

An oil phase containing 5 g of FT wax was added to the aqueous phase containing 0.2 g of surfactant mixture (SDS and Triton X-100 in a 4:1 mass ratio) in 45 mL of deionized water and heated to 98 °C with stirring at 400–500 rpm until completely melted. The mixture was then homogenized using a Branson Digital Sonifier 450 (Branson Ultrasonics Corp., Danbury, CT, USA) at 700 W output power for 10 min in pulse mode (pulse 3 s, pause 2 s). The resulting emulsion was diluted twofold with a 0.1% aqueous PVA solution and additionally stirred at 400–500 rpm for 30 min at ambient temperature. Upon this, the resulting suspension was filtered and washed with deionized water 3 times.

#### 2.2.2. Encapsulation of FT Wax in a Melamine Formaldehyde Shell

The formation of the polymeric shell was performed with in situ polymerization of melamine formaldehyde (MF) resin around the FT wax particles. To do this, a methylolmelamine prepolymer aqueous solution was prepared first. The methylolmelamine prepolymer was prepared while maintaining a melamine concentration not exceeding 50 mg/mL in the liquid phase to prevent spontaneous polymerization of the melamine–formaldehyde resin. In particular, an appropriate portion of formaldehyde solution (Table 1) was added to the aqueous phase under moderate stirring (400–500 rpm). Afterward, the pH was adjusted to 9 with TEA, and the mixture was heated to 60 °C. Next, the melamine was added stepwise to avoid a local oversaturation of prepolymer solution. The temperature was set to 65 °C, and the mixture was vigorously stirred (800–900 rpm) at least for 30 min until the transparent methylolmelamine prepolymer solution was formed. Various core-to-monomer (C/M) mass ratios of FT wax (core) and melamine–formaldehyde (monomers) were used (Table 1). The melamine/formaldehyde molar ratio was 1/8. Furthermore, for C/M = 2, the melamine/formaldehyde molar ratio of 1/3.5 was additionally used.

The polymerization was initiated by adjusting the pH of the FT wax suspension to 4.5–5 by 5 wt.% H_2_SO_4_ aqueous solution. Next, the as-prepared prepolymer solution was added dropwise with a volume flow rate of 0.1 mL/min under gentle stirring. The pH was maintained in the 4.5–5 range. Upon prepolymer addition, the temperature was set for 50 °C and the polymerization was carried out for 3 h. Finally, the reaction was stopped by pH neutralization. The resulting FT/MF capsules were rinsed with a hot (50 °C) water/ethanol (50/50 vol.) mixture for gently removing unencapsulated FT wax from the capsule suspension and dried at 60 °C overnight.

#### 2.2.3. Incorporation of FT/MF Capsules to Gypsum Plaster

The impact of FT/MF capsules on the heating and cooling rate of gypsum plaster was investigated by adding 15 wt.% of the capsules to dry gypsum powder, followed by mixing with water according to the manufacturer instructions. The resulting plaster was applied onto wallboards to form a 2 cm thick layer. K-type thermocouples (PT100) were embedded in the middle of the plaster layer (1 cm from the surface) and at the interface between the wallboard and the plaster (2 cm from the surface) to monitor temperature changes during heating and cooling. The excess of the hardened plaster was further used for morphological and latent heat capacity studies.

### 2.3. Characterization and Analysis

#### 2.3.1. Fractional Composition of FT Wax

The fractional composition of raw FT wax was determined by simulated distillation (ISO 3405 [23]) using a CHROMOS GC-1000 (Chromos Engineering, Dzerzhinsk, Russia) gas chromatograph equipped with a flame ionization detector and an MXT-1HT SimDist capillary column (Restek Corporation, Bellefonte, PA, USA).

#### 2.3.2. Size Distribution

The size distributions of the FT wax particles and FT/MF capsules were determined using a Mastersizer 3000 laser diffraction analyzer (Malvern Panalytical, Malvern, UK). The mean particle diameter (Dv 50) was taken as the average particle size. Standard deviation was calculated based on 5 parallel measurements.

#### 2.3.3. Chemical Composition

The formation of the MF shell and successful FT wax encapsulation were verified using a Nicolet iS10 Fourier transform infrared (FTIR) spectrometer equipped with a Ge ATR crystal (Thermo Scientific, Waltham, MA, USA). Spectra were recorded in absorbance mode over the wavenumber range of 4000–600 cm^−1^ as an average result of 32 iterative scans.

#### 2.3.4. Morphological Analysis

The morphology of the MF/FT capsules was characterized using a JEOL SEM FIB JIB-4501 scanning electron microscope (JEOL, Tokyo, Japan). Capsule suspensions were drop-cast onto aluminum stubs covered with conductive carbon tape and allowed to dry under ambient conditions. The samples were then sputter-coated with a 5 nm thick layer of gold to enhance conductivity. SEM images were acquired using an accelerating voltage of 10 kV.

#### 2.3.5. Thermal Stability

The thermal stability of the MF/FT capsules was assessed by thermogravimetric analysis (TGA) using an STA 449 F5 Jupiter instrument (Netzsch, Selb, Germany). Measurements were performed under a nitrogen atmosphere with a heating rate of 10 °C/min over a temperature range of 40 to 800 °C. Approximately 10 mg of sample was used for each analysis to obtain thermal decomposition profiles. From the TG curves, the onset temperature (T_on_) and endpoint temperature (T_off_) of each decomposition step were determined as described in ISO 11358-1:2022 [24]. For multi-stage decomposition without a plateau between steps, the tangent at the point of minimum gradient was applied to define the onset and endpoint temperatures of the intermediate decomposition stages. Derivative thermogravimetric (D-TGA) curves were obtained by differentiation of the TG curves, and the temperature at the maximum decomposition rate (MRDT) was established.

#### 2.3.6. Latent Heat Storage Properties

The latent heat storage properties of FT wax, FT/MF capsules, and gypsum plaster with the addition of FT/MF capsules were studied with differential scanning calorimetry (DSC) using a DSC 214 Polyma (Netzsch, Selb, Germany) instrument. The heating and cooling curves were acquired in the −50 to 120 °C range with a ramp of 10 °C/min. The sample mass was 5 mg for FT/MF capsules and 10 mg for FT wax and the gypsum plaster. The collected DSC curves from three runs were time-integrated to figure out a stored and released latent heat (enthalpy) as an area of endothermic (ΔH_m_) and exothermic (ΔH_s_) peaks, respectively. The apparent enthalpy-based loading efficiency (E_a_) was calculated based on the mean ΔH_m_ and ΔH_s_ values.

The temperature ranges of latent heat storage and release of FT wax, FT/MF capsules, and plaster with FT/MF capsules were specified with the geometric construction method defined in ISO 11357-1:2023 [25]. In particular, the raw DSC curves were smoothed using the Loess method with span values of 0.02 to 0.05 to preserve the inflection points of the main DSC peaks. To identify the inflection points on the leading and trailing edges of each peak, the first derivative of heat flow with respect to temperature was calculated; the derivative curve was then smoothed in the same manner and plotted on a secondary Y axis. The first valuable maximum positive derivative was used to locate the inflection point on the leading edge of the endothermic peak, whereas the last valuable maximum negative derivative was used to locate the inflection point on its trailing edge. The baseline was defined by the zero crossings of the first and second derivatives and constructed by fitting the selected anchor points with a Boltzmann function. A tangent was then drawn at the leading-edge inflection point, and the intersection of this tangent with the extrapolated baseline was determined and reported as T_m_on_. In an analogous manner, a tangent was drawn at the trailing edge inflection point, and the intersection with the post-transition baseline was determined and reported as T_m_off_. The same procedure was applied to determine the onset and offset temperatures of the exothermic peak, reported as T_s_on_ and T_s_off_, respectively.

#### 2.3.7. Evaluation of FT Wax Leakage

The stability of the FT/MF capsules was assessed with a leakage test. To do this, approximately 100 mg of the capsules were placed onto cellulose filter paper and heated in the drying box at 110 °C for 24 h. Upon heating, the mass of the capsule samples was measured again, and the relative leakage rate was calculated. An average leakage rate and standard deviation were calculated as a result of three independent measurements.

#### 2.3.8. Analysis of the Effect of FT/MF Capsules on Heating and Cooling of Gypsum Plaster

For thermal analysis, the plaster-coated wallboard with embedded FT/MF capsules was irradiated with infrared light for 30 min and then cooled at 3 °C for an additional 30 min. The heating was performed with a commercially available IR lamp with a nominal power of 250 W and irradiating in the visible red and near IR range (up to 2500 nm) according to manufacturer specifications. The heating rate was adjusted by varying the distance between the lamp and plaster surface. The irradiance at the plaster surface was estimated using the inverse square law approximation for a point source as

(1) $$E=P/\left(4\pi d^{2}\right)$$

where P is the lamp nominal power (250 W) and d is the distance from the lamp to the plaster surface (15 cm, 20 cm, and 25 cm). The estimated irradiance values were 884 W/m^2^ at 15 cm, 497 W/m^2^ at 20 cm, and 318 W/m^2^ at 25 cm. The temperature within the plaster layer was monitored with the embedded PT 100 thermocouples (FonLab, Saratov, Russia) using an automated data acquisition system. The resulting heating/cooling curves were derived from three experimental runs. The heating/cooling curves were processed with standard software to calculate the heating/cooling rate curves as a first-order derivative.

## 3. Results and Discussion

### 3.1. Composition and Thermal Properties of the FT Wax

**Figure 1:**
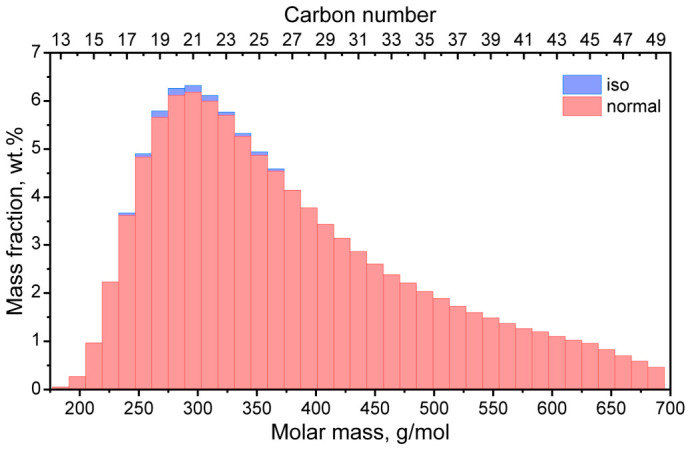
Molar mass/carbon number distribution histogram of the FT wax.

At the first step, the composition of the FT paraffin wax was studied with simulated distillation gas chromatography. The FT wax had a monomodal molar mass distribution with the maximum corresponding to C20–C22 (280–310 g/mol) alkanes and a broad shoulder extended toward high molecular mass fractions (Figure 1). Overall, the FT wax consisted of C13–C49 alkanes. The main fraction was n-alkanes (99.09 wt.%), which is inherent to waxes obtained through the Fischer–Tropsch process [26]. Iso-alkanes were a very minor fraction and belonged to C17–C28 components.

**Figure 2:**
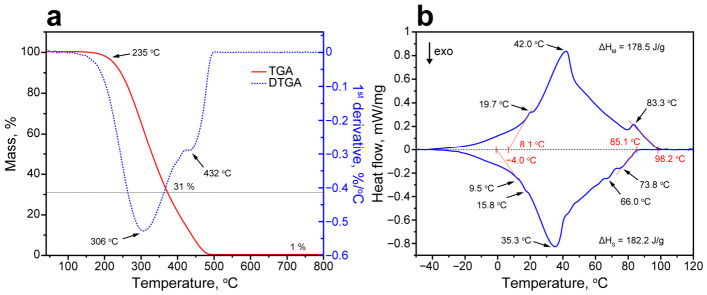
(**a**) TGA and D-TGA curves of FT wax and (**b**) DSC melting (top) and solidification (bottom) curves of FT wax.

The thermal decomposition profile of the initial FT wax (Figure 2a) reveals two distinct stages that directly correlate with its compositional heterogeneity. The first stage, representing the predominant low-molecular-mass fraction (~69 wt.% of total wax, as determined by residual mass analysis), initiates at 235 °C, which was established by the tangent to the TGA inflection point at the maximum decomposition rate of 305 °C. The endpoint of the first stage was at 365 °C with a 31 wt.% residue. This primary decomposition aligned with the C13–C25 n-alkane components that dominate the molar mass distribution (main mode at C20–C22), undergoing random chain fission typical of paraffin pyrolysis under inert conditions. The secondary stage, attributed to the higher-molecular-mass shoulder (~30 wt.%, C26–C49 n-alkanes), revealed a less pronounced D-TGA peak adjacent to the minimum-gradient inflection, with a maximum decomposition rate at 432 °C and the endpoint at 471 °C. The mass residue was ~1 wt.%. The overlap of these stages, characteristic of broad-cut synthetic waxes, precludes unambiguous secondary onset delineation with the conventional tangent methods; however, mass balance closure allows for quantifying the contribution of the high-molecular-weight fraction.

The phase change behavior of the FT wax was studied with DSC (Figure 2b). The melting curve of the FT wax revealed a wide endothermic peak with an onset melting temperature (T_m___on_) of 8.1 °C and an offset melting temperature (T_m_off_) of 98.2 °C. The main peak melting temperature was 42.0 °C, which is consistent with the monomodal FT wax component distribution. The position of the major peak can be attributed to the melting of the FT wax components with the highest mass content (C20–C22). The partially resolved peaks were found at 19.7 °C and 83.3 °C. The low-temperature peak was consistent with the melting temperatures of C16 and C17 components (18.1 °C and 21.8 °C, respectively) [27,28]. Gas chromatography analysis identified these low-molecular-weight components as the first substantial mass fraction within the FT wax. In turn, the high-temperature peak can be assigned to the melting of high-molecular fractions [29]. The total melting enthalpy ΔH_m_ was 178.5 J/g. The solidification curve revealed a wide exothermic peak as well, with the onset solidification temperature (T_s___on_) of 85.2 °C and offset solidification temperature (T_s_off_) of −4.0 °C. The main peak solidification temperature was 35.3 °C. Additionally, the solidification curve revealed a shoulder extended toward a high-temperature range that can be related to the solidification of high-molecular factions and the formation of metastable n-alkane phases prior to the formation of stable crystalline ones. The minor peaks at 9.5 °C and 15.8 °C were due to the solidification of low-molecular-weight components. The total solidification enthalpy (ΔH_s_) was 182.2 J/g. The measured latent heat capacity of the FT wax is consistent with the values reported previously for the waxes with the similar composition [30] and comparable with the capacity of individual paraffins [27].

### 3.2. Synthesis and Characterization of FT/MF Capsules

**Figure 3:**
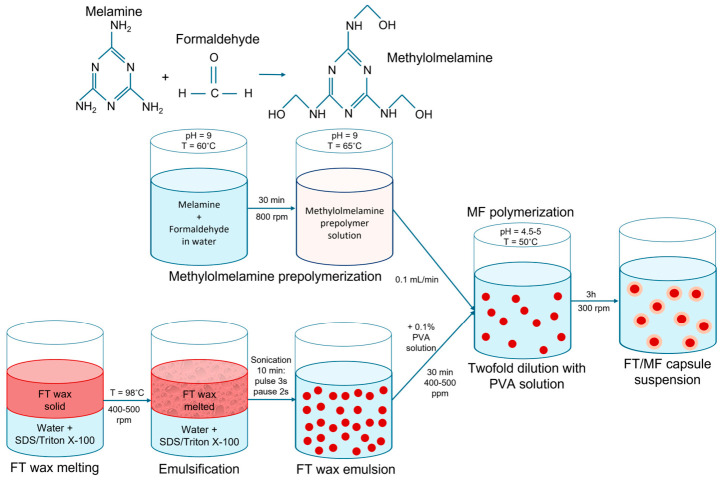
The schematic illustration for the synthesis of FT wax capsules with MF shell.

According to DSC, the FT wax was completely melted at 98.2 °C. This restricts available methods for preparation of emulsion and its further polymerization. The mechanical homogenization was not efficient, as it was complicated to maintain the constant temperatures of the premixed aqueous and oil phases and homogenizer tip. Additionally, the mechanical homogenization at 10,000–12,000 rpm resulted in excessive foaming of surfactants dissolved in the heated aqueous phase. Thus, an ultrasonic (US) homogenization was chosen for emulsion preparation. The US tip can be easily preheated with emulsion, and it self-maintains the temperature while the US is operational. Furthermore, the heating did not allow for employment of polymerization reactions initiated below 98 °C, like polyurethane formation, and therefore excluded the addition of any monomers to aqueous or oil phases prior to the preparation of emulsion. Thus, the general strategy for encapsulation of FT wax involved the following steps: the preparation of emulsion under the offset temperature of the FT wax melting; cooling; and the formation of a polymeric shell around the resulting dispersion particles under the suitable temperature by addition of water-soluble monomers. To fulfill this, the in situ polymerization of the melamine formaldehyde shell was chosen as an optimal approach. The scheme of FT/MF capsule preparation is shown in Figure 3.

**Figure 4:**
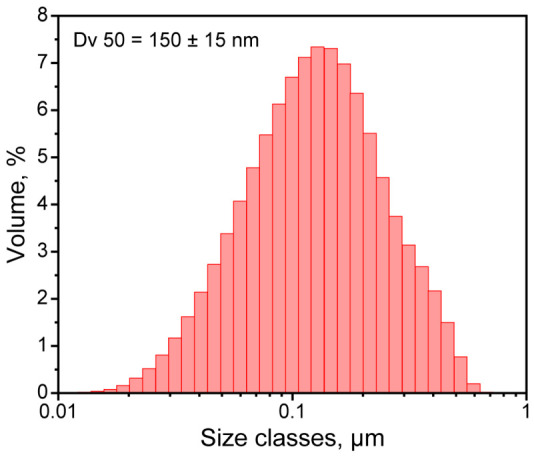
Size distribution of the PVA-stabilized FT wax suspension.

According to the experimental procedure, the portion of FT wax was added to the aqueous phase, and the mixture was heated and US-treated to prepare an emulsion. The mixture of SDS and Triton X-100 was chosen due to their synergistic interaction and leading to a condensed mixed interfacial monolayer, which enhances interfacial film cohesion [31]. The mass ratio of 4:1 was demonstrated to be optimal for stabilization of n-alkane emulsions and facilitation of MF polymerization [17]. Upon emulsification, the emulsion was twofold diluted with PVA solution, stirred for 30 min under heating, and finally cooled to the ambient temperature. PVA was used as a protective colloid to improve the colloidal stability of the resulting dispersion and to promote the uniform deposition and homogeneous polymerization of the melamine formaldehyde shell [17]. Figure 4 shows the particle size distribution of the resulting dispersion measured with laser diffractometry. The median particle size was 150 ± 15 nm, which is typical for ultrasonic homogenization.

The next step was the formation of a melamine formaldehyde shell using the FT wax suspension as a template. To do this, melamine and formaldehyde were prepolymerized in an alkaline medium at 65 °C. The formation of water-soluble methylolmelamine was indicated by the transparency of the solution. The different molar M/F ratios resulted in different prepolymer products. At an M/F molar ratio of 1/8, the formaldehyde is in a considerable excess so that the methylolation predominantly occurs at the initial stage of the reaction. Highly methylolated melamine derivatives are formed, including hexamethylolmelamine [32]. Condensation reactions between methylol groups at this stage are weak, and the intermediates are predominantly linear or slightly branched and highly soluble. At a molar ratio of 1/3.5, the excess of formaldehyde is significantly lower, which limits the degree of methylolation. Under these conditions, condensation reactions between methylol and amine groups begin to proceed more rapidly, forming methylene and methylene ether bridges. The intermediates are characterized by a lower degree of methylolation, a higher degree of branching, and a tendency to form a spatial network already in the early stages of polymerization [33].

Afterwards, the prepared prepolymer solution was dropwise added to the FT wax dispersion while maintaining its acidic pH. In acidic conditions and under moderate heating, the condensation of methylolmelamine prepolymer is initiated, which leads to the formation of methylene ether bridge linkages, which are further transformed into methylene linkages by formaldehyde elimination [18]. Thus, the high content of methylol groups provides a large number of reactive centers, which give rise to the formation of a more uniform spatial network during final condensation. In turn, the lower content of methylol groups results in a branched polymeric network due to early branching at the prepolymerization step. As the condensation was ongoing, the solubility decreased with the molecule growth, and the polymer migrated from the volume of the aqueous phase to the interface. In this way, the deposition and final condensation of the polymer form the melamine formaldehyde shell.

**Figure 5:**
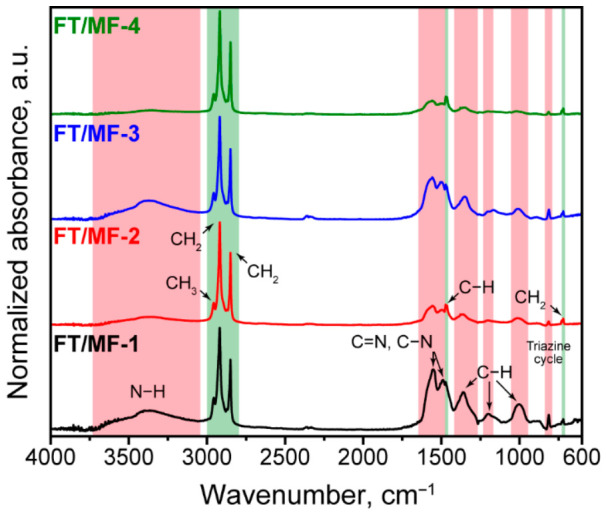
FTIR absorption spectra of FT/MF capsules: the red lines correspond to the MF shell, and the green lines correspond to vibrations of FT.

The successful formation of the MF shell and encapsulation of the FT wax was confirmed with FTIR spectroscopy (Figure 5). The FTIR spectra of the FT/MF capsules revealed the maximums of adsorption inherent to paraffins (green lines) and FM resin (red lines), respectively. In particular, paraffins can be distinguished by antisymmetric stretching of CH_3_ groups at 2956 cm^−1^, symmetric and antisymmetric stretching of CH_2_ groups at 2917 and 2849 cm^−1^, C–H stretching at 1471 cm^−1^, and CH_2_ rocking vibration at 720 cm^−1^ [34]. In turn, the wide band around 3330 cm^−1^ was due to stretching vibrations of primary and secondary N–H groups; the peak at 1552 cm^−1^ corresponded to ring vibration of the C=N group; the peaks at 1489, 1362, and 1005 cm^−1^ were due to vibrations of C–H groups, and the peak at 813 cm^−1^ was due to vibration of the triazine cycle, which is inherent to MF polymer [33,35]. Furthermore, the increase in the C/M ratio in FT/MF capsules was attended by a reduction in the absorbance intensity of the MF shell with respect to that of the FT core, which suggested the increase in the loading efficiency of the capsules. Comparing the capsules prepared with the same C/M ratio, the capsules with an M/F molar ratio of 1/3.5 demonstrated a higher intensity of absorbance due to the MF shell compared to the capsules with an M/F ratio of 1/8, which can be related to an increase in initial melamine content in the system.

**Figure 6:**
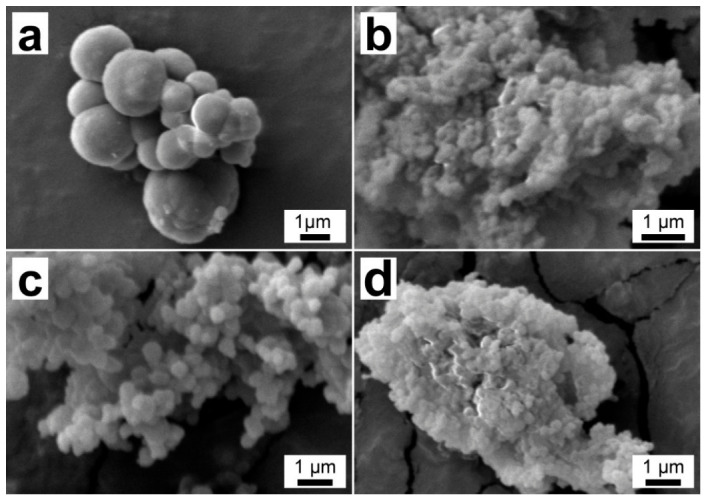
SEM images of FT/MF capsules: (**a**) FT/MF-1; (**b**) FT/MF-2; (**c**) sample FT/MF-3; and (**d**) FT/MF-4.

The morphology of the FT/MF capsules was studied with SEM (Figure 6). In the microscale, the capsules demonstrated different morphology depending on the C/M ratio. The increase in the polymer content resulted in the growth of the capsule size, which suggests the formation of a thicker capsule shell. Furthermore, the morphology of the capsules with C/M = 1 suggested the inclusion of several FT cores within a polymeric shell. The capsules with C/M = 2 and an M/F molar ratio of 1/3.5 revealed a nanoscale size and the lowest degree of aggregation, which can be related to less free formaldehyde available in the system.

**Figure 7:**
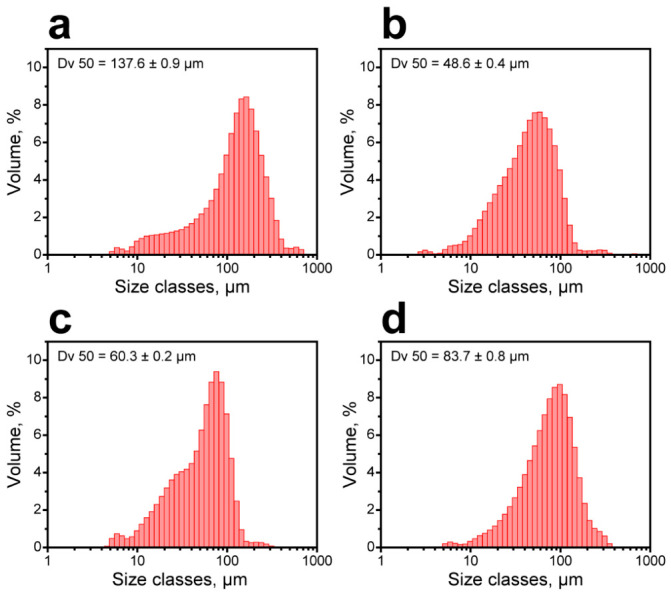
Size distribution of the FT/MF capsule powders: (**a**) FT/MF-1; (**b**) FT/MF-2; (**c**) FT/MF-3; and (**d**) FT/MF-4.

In the macroscale, the capsule aggregation was evaluated with laser diffraction. Figure 6 shows the size distribution of the FT/MF capsules redispersed in aqueous medium after drying (Figure 7). Compared to the initial FT wax suspension, the mean particle diameter Dv 50 increased by 2–3 orders of magnitude, from hundreds of nanometers to tens and hundreds of micrometers. Apparently, the capsule aggregation may occur due to spontaneous growth of the temperature in the system. As was demonstrated by Merline et al. [18], the condensation of methylolmelamine prepolymer is exothermic. In the particular synthesis, the temperature tended to spontaneously increase to around 50 °C. In case the target condensation temperature was set to 45 °C, the reaction mixture was overheated by 6 °C. If the target temperature was set to 48 °C, the reaction mixture was overheated by 9 °C, which suggested more intensive methylolmelamine condensation. The temperature growth resulted in condensation of MF resin in the volume of the aqueous phase along with the interfacial condensation. It should be noted that despite the significant aggregation compared to the initial FT wax suspension, the resulting dry powders of FT/MF capsules still demonstrated a relatively low mean size from 48.6 ± 0.4 to 137.6 ± 0.9 µm and can be easily embedded into conventional building materials like plasters and paint layers.

**Figure 8:**
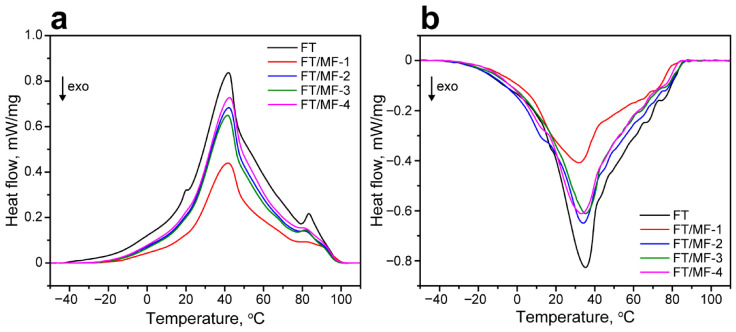
(**a**) DSC melting curves of FT wax and FT/MF capsules. (**b**) DSC solidification curves of FT wax and FT/MF capsules.

The latent heat storage properties of MF/FT capsules were studied with DSC. Figure 8a shows the comparison of melting curves of the initial and encapsulated FT wax. The encapsulated FT wax demonstrated the similar shape and position of the main endothermic peak as the initial one. The DSC curves of FT/MF capsules did not reveal the low-temperature peak assigned to the melting of the low-molecular-weight FT wax fraction, while the high-temperature peak assigned to the melting of the high-molecular wax components reduced and transformed due to elongation of the main peak. Apparently, this can be related to the wax nanoconfinement and effect of the polymeric shell. The melting enthalpy ΔH_m_ reduced along with the C/M ratio due to a decrease in FT wax content in the capsules. The solidification curves in Figure 8b demonstrated the similar position of the exothermic peaks as well, with the solidification enthalpy ΔH_s_ reduced along with the C/M ratio. The data on the onset, peak, and offset phase transition temperatures along with melting and solidification enthalpies of the FT wax and FT/MF capsules are summarized in Table 2.

Considering the figured-out values of melting and solidification enthalpies of bare and encapsulated FT wax, one can calculate its apparent enthalpy-based loading efficiency as

(2) $$E_{a}=\frac{∆H_{m/caps}+∆H_{s/caps}}{{∆H}_{m/FT}+{∆H}_{s/FT}}\times 100\%,$$

where ΔH_m/caps_ and ΔH_s/caps_ are melting and solidification enthalpies of the encapsulated FT wax, whereas ΔH_m/FT_ and ΔH_s/FT_ are those of the initial FT wax [36].

The theoretical content can be calculated as

(3) $$E_{t}=\frac{m_{FT}}{m_{FT}+m_{M}}\times 100\%,$$

where m_FT_ is the mass of the FT wax (5 g) and m_M_ is the mass of the monomers (melamine and formaldehyde) employed for shell polymerization [37]. The calculated E_a_ and E_t_ values are given in Table 2. The enthalpy-based loading efficiency was directly related to the C/M ratio and exceeded the theoretical one based on the mass balance. One possible explanation is the effect of the MF shell, which potentially may have several DSC interferences with the FT wax related to moisture evaporation, demethylation, and crosslinking condensation [18]. The moisture evaporation is endothermic and occurs in the 100–180 °C range. The demethylation is exothermic and tends to take place in the 140–160 °C range. Finally, the crosslinking condensation occurs at temperatures above 160 °C and is exothermic as well. Among these, only the temperature range of moisture evaporation may slightly overlap with that of FT/MF capsules. However, according to the measured DSC curves, the endpoint of the endothermic peak of FT/MF capsules was 80.0–85.8 °C. Even though moisture evaporation began below 100 °C, the overlap was minimal and therefore could not contribute substantially to the endothermic peak area. Thus, it is more likely that a portion of the added methylolmelamine was neither adsorbed onto the FT surface nor incorporated into the capsule shell and therefore did not contribute to the final mass of the FT/MF capsules. This interpretation is also supported by the observed temperature increase in the system, which suggests that methylolmelamine underwent bulk condensation, including polymerization on the beaker walls.

**Figure 9:**
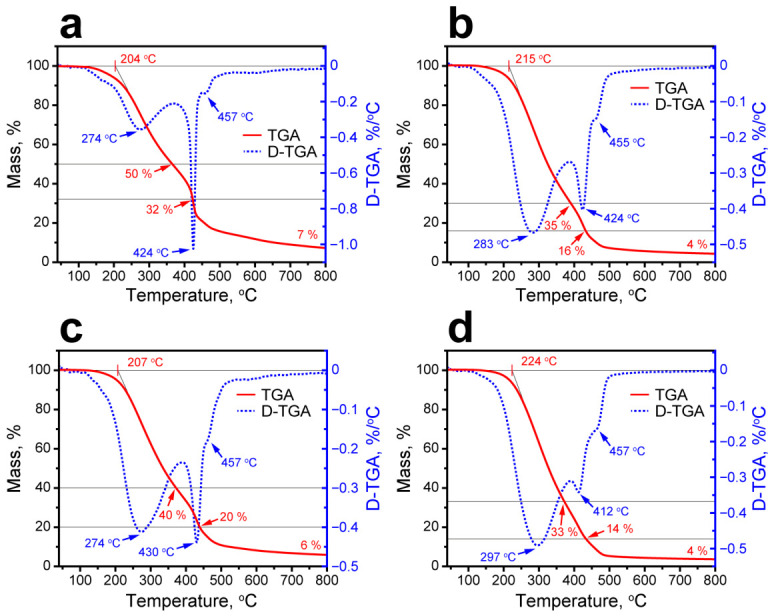
TGA and D-TGA curves of FT/MF capsules: (**a**) FT/MF-1; (**b**) FT/MF-2; (**c**) FT/MF-3; and (**d**) FT/MF-4. The black lines indicate the midpoints between decomposition stages.

The thermal stability of the MF/FT capsules was evaluated with TGA and D-TGA (Figure 9). For FT/MF-1 capsules, the first true minimum in the D-TGA curve appeared at 274 °C, corresponding to the maximum decomposition rate (MRDT), and the tangent drawn at this point to the TGA curve gave an onset temperature of 204 °C (Figure 9a). This stage ended at 317 °C and was assigned primarily to the condensation of the MF shell, including the elimination of formaldehyde from ether bridges and the formation of more stable methylene linkages [18]. The TGA curve did not show a clear plateau between the first and second stages; thus, the residual mass (m_r_) after the first stage was estimated as the midpoint between the mass at the end of the first stage and the beginning of the second, which gave about 50 wt.%. A weak shoulder in the D-TGA curve in the 115–165 °C range indicated a minor mass loss of about 2 wt.%, which can be attributed to evaporation of bound water released during self-condensation of methylol groups [18].

The second major stage began at 415 °C and ended at 431 °C, with the residual mass decreasing to 32 wt.%. This stage can be associated with cleavage of methylene bridges in the MF network. The third stage starts at 454 °C and continues gradually up to 800 °C, leaving about 7 wt.% residue. This final mass loss is consistent with degradation of the triazine ring structure [18]. Importantly, decomposition of the encapsulated FT wax proceeds concurrently with the degradation of the MF shell, so the observed thermal profile reflects the overlapping contributions of both core and shell materials.

TGA and D-TGA curves for the remaining FT/MF capsules (Figure 9b–d) were analyzed analogously. The comparison of the characteristic temperatures of thermal decomposition of FT wax and FT/MF capsules is given in Table 3.

Compared to the bare FT wax (T_on_ = 235 °C), all FT/MF capsules exhibited lowered initial decomposition temperatures, primarily due to low-temperature mass loss from bound water evaporation in the MF shell (~115–165 °C) and formaldehyde elimination from ether bridges (204–224 °C). The subsequent FT wax decomposition range also shifts downward, which can be attributed to the Gibbs–Thomson effect arising from nanoscale core confinement within the MF shell. Nevertheless, the capsules maintained sufficient thermal stability below 200 °C, which well exceeded their operational latent heat storage range (8–98 °C) derived from DSC and confirmed their suitability for thermal regulation under typical building thermal cycles.

The stability of the FT/MF capsules was evaluated by heating for 24 h in a drying box at 110 °C. Although under real building operation conditions the heating of interior surfaces is not intended to exceed 35 °C (or 50–60 °C, occasionally), such a harsh heating ensured the FT wax core was completely melted and allowed for accelerated stability assessment of FT/MF capsules. Upon heating, the leakage rate was calculated by relative mass reduction in the capsules as

(4) $${LR}_{m}=\frac{m_{0}-m_{i}}{m_{0}}\times 100\%,$$

where m_0_ is the initial mass of the capsules, and m_i_ is the capsule mass after heating. The results are summarized in Table 4. Although the average mass loss varied from 18.4 ± 1.2 wt.% to 22.6 ± 1.1 wt.% depending on the C/M ratio, the capsules did not reveal any visible traces of FT leakage on the paper filter. Therefore, the mass loss can be related to the evaporation of the bound water during MF self-condensation and residues of unreacted formaldehyde. This suggestion is confirmed by higher mass loss by the FT/MF capsules prepared with higher formaldehyde addition (FT/MF-1 and FT/MF-2).

### 3.3. FT/MF Capsules as a Thermoregulating Additive to Commercial Gypsum Plaster

**Figure 10:**
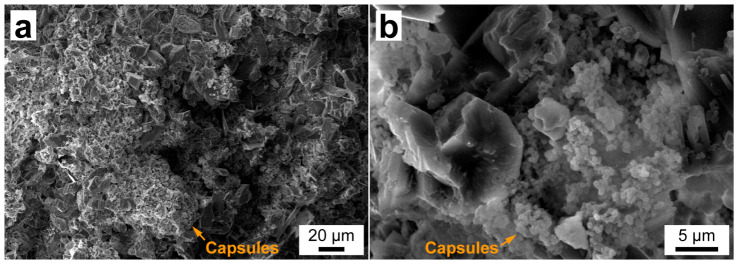
(**a**) SEM image of gypsum plaster with FT/MF-3 capsules taken at 450× magnification; (**b**) SEM image of gypsum plaster with FT/MF-3 capsules taken at 3k× magnification.

Taking into account the results on the structure, thermoregulating properties, and stability of the FT/MF capsules, sample FT/MF-3 was chosen for further evaluation as a thermoregulating additive to gypsum plaster. Figure 10 shows the SEM images of the cross-section of the hardened plaster layer prepared with the addition of FT/MF capsule powder to the initial dry mix. The capsules are well dispersed within the plaster volume, yet they appear aggregated; that may be a consequence of the manual mixing (Figure 10a). The hardening did not affect the shape and morphology of the single capsules, which confirms their good stability in the plaster layer (Figure 10b).

**Figure 11:**
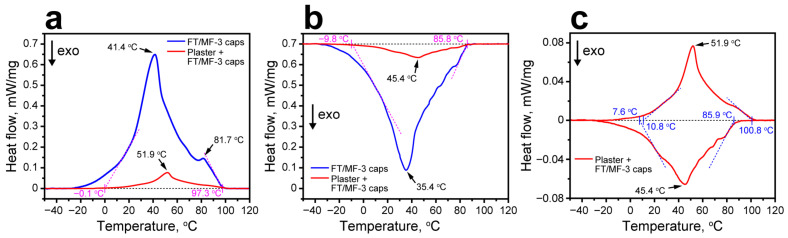
(**a**) Comparison of DSC heating curves of FT/MF-3 capsules and the plaster with the addition of FT/MF-3 capsules. (**b**) Comparison of DSC cooling curves of FT/MF-3 capsules and the plaster with the addition of FT/MF-3 capsules. (**c**) DSC curves of the plaster with the addition of FT/MF-3 capsules with the temperature ranges for accumulation and release of thermal energy.

The latent heat storage properties of the plaster with the addition of FT/MF-3 capsules were studied with DSC. Addition of the FT/MF-3 capsules gave rise to wide endothermic and exothermic peaks on DSC curves of hardened plaster (Figure 11a,b). Confinement of FT/MF-3 capsules in a thermally inert mineral matrix resulted in additional broadening and peak temperature shifts due to restricted heat transfer (Figure 11c). Geometric construction via extrapolation of the linear sections flanking the main endothermic peak in the plaster heating curve defines the heat storage range from 10.8 ± 1.6 °C to 100.8 ± 1.2 °C, corresponding to the most efficient thermal energy accumulation. In turn, the extrapolation of the linear sections of the exothermic peak suggests the most efficient latent heat release in the range from 7.6 ± 0.3 to 85.9 ± 0.9 °C. The endothermic peak area corresponded to 20.5 ± 3.5 J/g, while the exothermic peak area was 21.3 ± 3.3 J/g. According to Equation (2), this corresponded to enthalpy-based loading efficiency in the plaster of 13.3%, which is in good agreement with the initially intended content of 15.0%.

**Figure 12:**
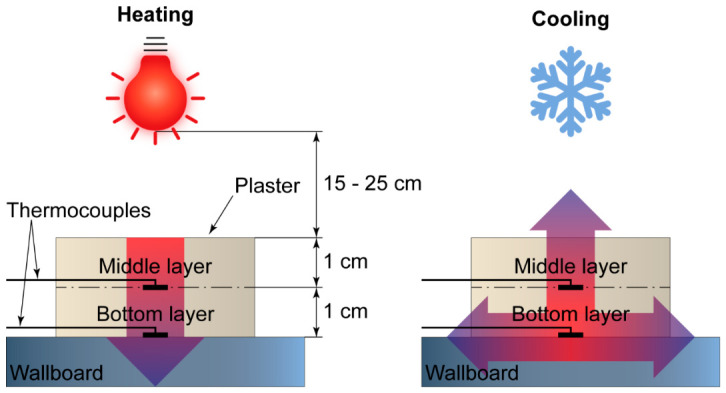
The scheme of the experiment on the study of the effect of FT/MF-3 capsules on the heating/cooling rate of the plaster layer. Wide arrows show the direction of the temperature gradient during heating and cooling.

Finally, the effect of FT/MF-3 capsules on the heating and cooling rates of the plaster layer was studied by measuring the temperature within the layer under external heating with an IR lamp. The scheme of the experiment is shown in Figure 12. The plaster layer with embedded thermocouples was placed under an IR lamp at various distances (15–25 cm) to heat the plaster with various heating rates designated as “fast” at a distance of 15 cm, “moderate” at a distance of 20 cm, and “slow” at a distance of 25 cm. Upon the heating cycle, the plaster was cooled in a chilling chamber.

**Figure 13:**
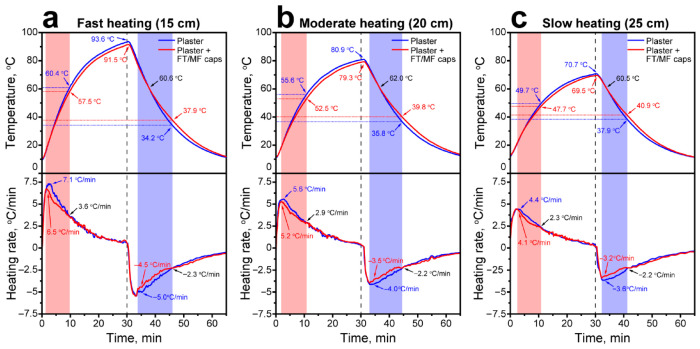
The heating/cooling curves of the middle plaster layer (on top) and corresponding heating/cooling rates (on bottom) acquired after fast (**a**), moderate (**b**), and slow (**c**) heating. The dotted line separates heating and cooling cycle.

Figure 13 shows the acquired heating and cooling curves as well as the corresponding heating/cooling rates calculated as the first-order derivatives for the middle layer of the plasters. The temperature of the plaster with the addition of the FT/MF-3 capsules was consistently lower than the temperature of the control plaster without capsule addition. Under a fast heating rate (Figure 13a), the control sample heated up to 93.6 °C in 30 min, whereas the sample with containers heated up to 91.5 °C. At the initial stage of the heating, the heating rate of the control plaster increased to 7.1 °C/min and then gradually decreased to 0.6 °C/min. For the plaster with containers, the heating rate increased to 6.5 °C/min and gradually decreased to 0.7 °C/min. This was quite different from plasters containing additives based on the individual paraffin, in which the heating rate leveled off around the phase transition temperature and sharply increased as the phase transition had been passed [38]. The heating rates equalized at 3.6 °C/min. At this point, the temperature difference between the plasters was 2.9 °C and only slightly reduced at the end of the heating.

In the cooling cycle, the cooling rate of the control plaster was 5.0 °C/min (as an absolute value), whereas the cooling rate of the plaster with the addition of FT/MF-3 capsules was 4.5 °C/min. The cooling rates equalized at 2.3 °C/min. At this point, the temperature difference between the plasters was 3.7 °C. The plaster with the addition of FT/MF-3 capsules preserved a higher temperature, although at the initial stage of the cooling, it was colder than the control one. This trend changed when the temperature of the plasters reached 60.6 °C, which apparently corresponds to the onset solidification temperature of the main FT fraction under the experimental conditions.

The reduction in heating rate did not lead to significant changes in the nature of heating and cooling of the plaster layers, except for a decrease in the maximum heating temperature of the layers and in the difference in heating and cooling rates (Figure 13b,c).

**Figure 14:**
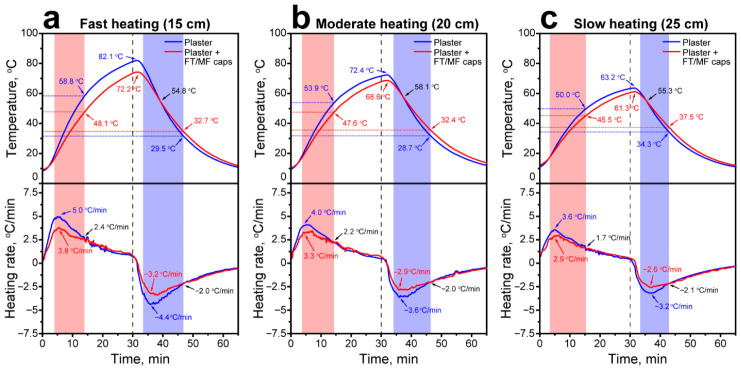
The heating/cooling curves of the bottom plaster layer (on top) and corresponding heating/cooling rates (on bottom) acquired after fast (**a**), moderate (**b**), and slow (**c**) heating. The dotted line separates heating and cooling cycle.

In turn, the increase in the layer thickness to 2 cm resulted in the more prominent difference in heating and cooling behavior of the plaster samples (Figure 14). For instance, at the fastest heating rate (Figure 14a), the control plaster heated up to 82.1 °C, whereas the plaster with FT/MF-3 capsule addition heated only to 72.2 °C in 30 min. The heating rate of the control plaster was 5.0 °C/min, while that of the plaster with the capsule addition was 3.8 °C/min. The heating rate equalized at 2.4 °C/min. At this point, the temperature difference between the plasters was 10.7 °C and only slightly reduced to the end of the heating to 9.9 °C.

In the cooling cycle, the cooling rate of the control plaster was 4.4 °C/min, while that of the plaster with the capsule addition was 3.2 °C/min. The heating rates equalized at 2.0 °C/min. At this point, the plaster with the addition of FT/MF-3 capsules cooled to 32.7 °C, and the control plaster cooled to 29.5 °C. The temperature difference was 3.2 °C. The further reduction in the heating rate of the plasters revealed the same trend: the control plaster heated and cooled faster compared to the plaster with FT/MF-3 capsules (Figure 14b,c).

Unlike the addition of the individual paraffins, the introduction of FT/MF capsules did not result in the appearance of prominent temperature plateaus in heating and cooling curves, which can be attributed to the sequential melting and solidification of FT wax components. On the other hand, these sequential phase transitions provide a smooth and gradual reduction in heating/cooling rates over a wide temperature range without abrupt changes as the phase transition of a single component is completed. Under the experimental conditions, the FT/MF capsules were effective in the accumulation of latent heat and slowing down the heating rate in the range up to 45.5–57.5 °C and in the release of the latent heat and slowing down the cooling rate in the range down to 29.5–37.9 °C, depending on the heating/cooling rate of the plaster layer and its thickness. This makes encapsulated FT wax a promising thermoregulating additive to dry building mixes. Further studies can be devoted to evaluation of the encapsulated FT wax effect on the air temperature alterations in the model experiments.

## 4. Conclusions

In this study, we have demonstrated the feasibility of utilizing a broad paraffin fraction obtained via the Fischer–Tropsch process as a versatile latent heat storage additive. The FT wax, comprising C13–C49 n-alkanes with 99.09% linear hydrocarbons, exhibited a wide phase transition range of 8.1–98.2 °C and a high latent heat capacity of 178.5 J/g, making it inherently suitable for thermoregulating applications across varying temperature conditions.

An effective encapsulation strategy was developed, employing ultrasonic emulsification followed by in situ polymerization of the melamine–formaldehyde resin around the FT wax nanodispersion. The FT/MF capsules with optimized core-to-monomer ratios demonstrated loading efficiencies up to 78%, latent heat storage capacities of 104.5–163.4 J/g, and thermal stability up to 204 °C. The capsules with an M/F molar ratio of 1/3.5 (FT/MF-3) exhibited the most favorable combination of structural properties, latent heat storage capacity, and stable thermoregulating performance. Incorporation of 15 wt.% FT/MF-3 capsules into commercial gypsum plaster resulted in homogeneous dispersion within the hardened matrix without compromising capsule integrity. DSC analysis confirmed the preserved phase-change behavior with effective latent heat accumulation in the 10.8–100.8 °C range and release in the 7.6–85.9 °C range. The thermal performance evaluation demonstrated that the FT/MF-containing plaster consistently maintained lower heating rates and higher cooling temperatures compared to control samples, with the effect becoming more pronounced in thicker layers (2 cm versus 1 cm). Unlike individual paraffin additives that produce distinct temperature plateaus, the broad paraffin fraction enabled smooth, gradual thermal regulation without abrupt transitions.

The encapsulated FT wax thus represents a promising and versatile thermoregulating additive for dry building mixes, offering the advantages of a broad operating temperature range, high latent heat capacity, and cost-effectiveness derived from wide paraffin fractions rather than purified individual alkanes. Future work should focus on evaluating the impact of this additive on indoor air temperature stabilization under realistic diurnal cycles and optimizing capsule formulations for specific climate conditions. Optimization of capsule preparation is also of interest for improving their dispersity and avoiding aggregation.

## Figures and Tables

**Table 1:** Sample designation and component ratios used to form the melamine–formaldehyde resin shell on the surface of FT wax suspension.

Sample	C/M Mass Ratio	Water, mL	Melamine, g	Formaldehyde, g	M/F Molar Ratio
FT/MF-1	1/1	34	1.72	3.29	1/8
FT/MF-2	2/1	17	0.86	1.64	1/8
FT/MF-3	2/1	27	1.36	1.14	1/3.5
FT/MF-4	4/1	8.5	0.43	0.82	1/8

**Table 2:** Thermal properties of FT wax and FT/MF capsules derived from DSC.

Sample	T_m_on_, °C	T_m_, °C	T_m_off_, °C	ΔH_m_, J/g	T_s_on_, °C	T_s_, °C	T_s_off_, °C	ΔH_s_, J/g	E_a_, %	E_t_, %
FT	8.1 ± 0.6	42.0 ± 0.1	98.2 ± 0.2	178.5 ± 3.2	85.2 ± 0.9	35.3 ± 0.1	−4.0 ± 0.3	182.2 ± 3.5	100	100
FT/MF-1	−0.7 ± 1.2	41.8 ± 0.1	100.2 ± 0.2	107.4 ± 3.8	80.0 ± 1.5	32.1 ± 0.2	0.5 ± 0.6	104.5 ± 3.6	62	49
FT/MF-2	0.2 ± 0.9	42.1 ± 0.2	97.8 ± 0.3	151.1 ± 2.9	83.3 ± 0.6	34.1 ± 0.2	−4.2 ± 0.6	147.5 ± 3.0	82	65
FT/MF-3	−0.1 ± 1.1	41.4 ± 0.3	97.3 ± 0.5	154.8 ± 3.5	85.8 ± 0.7	35.4 ± 0.2	−9.8 ± 0.3	159.4 ± 2.8	78	65
FT/MF-4	0.3 ± 1.1	42.5 ± 0.1	98.1 ± 0.2	162.1 ± 2.7	83.0 ± 1.0	33.3 ± 0.1	−7.1 ± 0.5	163.4 ± 4.1	84	78

**Table 3:** The characteristic temperatures and thermal decomposition stages of FT wax and FT/MF capsules.

Stage		FT Wax	FT/MF-1	FT/MF-2	FT/MF-3	FT/MF-4
1	T_on_, °C	235	204	215	207	224
	MRDT, °C	306	274	283	274	297
	T_off_, °C	365	318	331	330	343
	m_r_, %	31	50	35	40	33
2	T_on_, °C	365	415	412	416	403
	MRDT, °C	432	424	424	430	412
	T_off_, °C	471	431	455	457	457
	m_r_, %	1	32	16	20	14
3	T_on_, °C	-	454	455	457	457
	MRDT, °C	-	457	455	457	457
	T_off_, °C	-	800	800	800	800
	m_r_, %	-	7	4	6	4

**Table 4:** The data on the mass loss by FT/MF capsules after incubation at 110 °C.

Sample	Iteration 1			Iteration 2			Iteration 3			LR_m_, %
	m_0_, mg	m_i_, mg	LR_m_, %	m_0_, mg	m_i_, mg	LR_m_, %	m_0_, mg	m_i_, mg	LR_m_, %	
FT/MF-1	100.9	78.1	22.6	99.4	78.1	21.4	102.4	78.0	23.8	22.6 ± 1.1
FT/MF-2	99.6	79.2	20.5	101.0	79.4	21.4	98.2	78.9	19.7	20.5 ± 0.9
FT/MF-3	100.5	80.8	19.6	99.1	84.0	15.2	101.9	80.7	20.8	18.6 ± 1.0
FT/MF-4	102.5	84.9	17.2	104.1	83.7	19.6	101.0	82.4	18.4	18.4 ± 1.2

## Data Availability

The original contributions presented in this study are included in the article. Further inquiries can be directed to the corresponding author.
